# Effect of Supplementation With Selenium-Yeast on Muscle Antioxidant Activity, Meat Quality, Fatty Acids and Amino Acids in Goats

**DOI:** 10.3389/fvets.2021.813672

**Published:** 2022-01-25

**Authors:** Xing-Zhou Tian, Jia-Xuan Li, Qing-Yuan Luo, Xu Wang, Mei-Mei Xiao, Di Zhou, Qi Lu, Xiang Chen

**Affiliations:** ^1^Key Laboratory of Animal Genetics, Breeding and Reproduction in the Plateau Mountainous Region, Ministry of Education, College of Animal Science, Guizhou University, Guiyang, China; ^2^Testing Center for Livestock and Poultry Germplasm, Guizhou Agricultural and Rural Affairs Office, Guiyang, China

**Keywords:** selenium-yeast, antioxidant activity, meat quality, fatty acid, amino acid

## Abstract

The objective of this study was to observe the effects of selenium-yeast (SY) on growth performance, muscle antioxidant activity, meat quality, fatty acid and amino acid profiles in growing goats. A total of 18 Qianbei-pockmarked goats were assigned to three groups (six duplicates per group) by body weight (25.75 ± 1.75 kg; mean ± standard deviation) according to a completely randomized design: (1) basal diet (CON); (2) CON with 2.4 mg/kg SY (LS); and (3) CON with 4.8 mg/kg SY (HS). The results indicated that goats receiving SY did not show any differences (*P* > 0.05) in terms of dry matter intake, growth performance, or muscle chemical composition. In addition, dietary treatment did not affect (*P* > 0.05) the pH values (pH_45min_ and pH_24h_), percentage of water loss, drip loss, or cooking loss. The HS group showed a significant increase (*P* < 0.05) in the dressing percentage, eye muscle area and meat color, as well as muscle total antioxidant capacity, glutathione peroxidase and 2,2-diphenyl-1-picrylhydrazyl scavenging activity levels, whereas it showed a significant drop (*P* < 0.05) in shear force and muscle malondialdehyde levels relative to the control. Feeding 4.8 mg/kg SY led to a significant (*P* < 0.05) decrease in the levels of C8:0, C14:0, C15:0, C16:0, C17:0, C18:0, C20:0 and total saturated fatty acids, whereas it led to a significant (*P* < 0.05) increase in C15:1 in comparison with that of the control group. Goats receiving 2.4 mg/kg SY had significantly (*P* < 0.05) increased C16:1, C17:1, C18:1n7, C18:2n6, C18:3n3, C20:4n6, C22:1n9, and PUFA relative to the control group. Compared with the control group, the treatment groups had higher (*P* < 0.05) levels of C18:1n9, C22:4, and monounsaturated fatty acids. The inclusion of 2.4 mg/kg SY induced significant (*P* < 0.05) increases in 4-aminobutyric acid, glutamic acid and umami amino acid concentrations compared to the control. In addition, the feeding of 4.8 mg/kg SY had significantly higher (*P* < 0.05) muscle serine, valine, isoleucine, leucine, ornithine hydrochloride, methionine, and tyrosine levels than the control group. Collectively, Se supplementation in the diet did not affect growth performance, muscle chemical composition, whereas it could improve meat quality, muscle antioxidant activity, fatty acid and amino acid profiles in Qianbei-pockmarked goats. This showed that the optimal accession SY level was 4.8 mg/kg under the experimental conditions of this study.

## Introduction

When the body suffers from oxidative stress (OS), the amount of reactive oxygen species (ROS) is enhanced and the antioxidant capacity decreases, leading to the initiation of lipid peroxidation, which may cause DNA oxidative damage and abnormal protein expression (1). The development of antioxidants to alleviate OS is popular with many researchers (2). The most important and known biological effect of selenium (Se) is its antioxidative effect. Indeed, Se is an essential trace element that has many biological functions, especially in preventing lung cancer, carcinoma of the prostate and liver cancer. However, excessive or inadequate Se intake will lead to physiological disorders or diseases. Se deficiency or excess can lead to poisoning caused by gastrointestinal disorders, vomiting, nausea, and diarrhea, thereby leading to impaired physiological functions (3).

Supplementation of Se in ruminant feed is achieved with additives such as sodium selenite, sodium selenate and selenium-yeast (SY) *Saccharomyces cerevisiae*. Antunović et al. (4) showed that organic Se in the feed could be transferred to the muscle and organs, and organic Se had a better bioavailability than inorganic Se in fattening lambs. Notably, SY is a source of organic Se (mainly selenomethionine) because it can be absorbed and retained more readily than inorganic Se (5). The general requirement for Se in the diet of ruminants is 0.1–0.3 mg/kg, and the maximum tolerance of Se is 5 mg/kg (6). Vignola et al. (7) demonstrated that lambs receiving low levels (0.30 and 0.45 mg/kg SY) of SY had no differences in growth performance, feed-to-gain ratio, carcass or meat quality. Goats generally have a strong tolerance of Se, and SY at a high level (4.0 mg/kg) is a relatively safe Se supplement for goats (6).

Polyunsaturated fatty acids (PUFAs) are important for body health. However, ruminant products have been criticized for their saturated fatty acids (SFAs) may have adverse effects on human health, which has contributed to declining consumption (8). Se has been proven to have strong antioxidant activity, delaying the onset of muscle oxidation reactions (9). Hence, ruminant receiving SY had the ability to improve unsaturated fatty acid (UFA) profiles in ruminant products. Moreover, Se has the capability of preventing apoptosis initiated by ROS and it participates in the regulation of amino acid (AA) profiles (10). Therefore, adding Se to the goat diet can not only improve growth performance but also enhance immune responses and antioxidant activity (11). However, research on the influence of a high dose of SY on meat quality in goats is rather limited. Considering the above, we hypothesized that a high dietary dose of SY would increase *longissimus dorsi* (LD) muscle antioxidant activity, improving meat quality, muscle fatty acids (FAs) and AAs derived from goats. Accordingly, the current research was performed to observe whether supplementation with Se in the diet could affect the meat quality, muscle antioxidant activity, FA and AA of Qianbei-pockmarked goats.

## Materials and Methods

### Animals, Diets, and Experimental Design

The feeding trial was carried out at a commercial goat farm (Guizhou, China). Signs of Se toxicity usually start to appear at Se levels of 5–8 mg/kg DM in ruminants (12). Therefore, the goats were raised for 74 d, which included a preparation period of 14 d and a formal experimental period of 60 d. Eighteen Qianbei-pockmarked wether goats (a Guizhou native goat breed) were assigned to three groups (six duplicates per group) by body weight (BW, 25.75 ± 1.75 kg; mean ± standard deviation) according to a completely randomized design: (1) basal diet (CON); (2) CON with 2.4 mg/kg SY (LS); and (3) CON with 4.8 mg/kg SY (HS). The SY as part of the basal ration and was mixed in the concentrate, and then the concentrate was mixed with the roughage evenly. SY was obtained from Jiangsu Qianbo Bioengineering Co., Ltd., Jiangsu, China. The information of SY is: food additive production license No. SC20133062401397, the Se content of the SY is 2000 ppm, and the appearance is a yellow powder with a uniform fineness. During the experiment, all animals received water freely and were fed at 08:30 and 16:30 for *ad libitum* intake. The rations were made according to the National Research Council [(13); NRC, Table 1].

**Table 1:** Ingredients and nutrient composition of experimental diets (DM basis).

**Ingredients, %**	**Content**	**Chemical composition, %**	**Content**
Peanut vines	50.00	Dry matter	90.15
White distiller's grains	10.00	Crude protein	11.95
Soybean residues	10.00	Gross energy, kJ/g	16.33
Green hay	9.30	Neutral detergent fiber	42.74
Corn	16.00	Acid detergent fiber	28.61
Soybean meal	3.00	Ether extract	2.35
Mineral premix^a^	0.50	Ash	8.72
Vitamin premix^b^	0.50	Calcium	0.96
NaCl	0.50	Phosphorus	0.15
Limestone	0.20		
Total	100.00		

a *Vitamin premix was purchased Guangzhou Everrich Animal Health Co., Ltd. (Guangzhou, China), and contained the following vitamins per kilogram feed: 4000000 IU of vitamin A, 600000 IU of vitamin D, 25,000 mg of vitamin E, 7,000 mg of DL-methionine, and 5,000 mg of L-lysine*.

b *Mineral premix was obtained the Earth Animal Nutrition and Health Products Co., Ltd. (Chongqing, China), and contained the following minerals per kilogram feed: 1,300 mg of Cu, 1,000 mg of Fe, 1,575 mg of Zn, and 595 mg of Mn*.

### Chemical Composition

Approximately 100 g of base diet was collected once weekly and mixed after the end of the feeding trial, and 500 g of feed was collected and dried at 65°C in a vacuum oven for 72 h, ground and passed through a 1-mm sieve for further analysis. The chemical composition of dry matter (DM), crude protein (CP), ether extract (EE), ash, calcium (Ca), phosphorus (P) and gross energy (GE) were measured according to the Association of Official Analytical Chemists [(14); AOAC]. Neutral detergent fiber (NDF) and acid detergent fiber (ADF) were determined according to Van Soest et al. (15). Each sample was analyzed in triplicate.

### Growth Performance

Dry matter intake (DMI) was recorded as the intake of each group of goats every day, and BW was measured on the first day and the last day before the morning feeding to calculate the initial weight (IW) and final weight (FW). The following formulas were used to calculate the average net gain (ANG), average daily gain (ADG), and feed conversion ratio (FCR): ANG (kg) = FW (kg) – IW (kg); ADG (g/d) = ANG/74 × 1000; FCR = DMI/ADG × 100%.

### Antioxidant Activity

The LD homogenate was prepared as follows: chopped tissue and phosphate buffered saline (PBS) were added to the glass homogenizer (1:9), and the homogenate was broken by ultrasonication using a Bransonic® ultrasonic cleaner (Branson ultrasonics corporation, USA). Next, the supernatant was transferred to a 1.5 mL tube after centrifugation at 4,000 × g for 10 min at 4°C and stored at −80°C until further analysis. The total antioxidant capacity (TAC), superoxide dismutase (SOD), glutathione peroxidase (GSH-Px), catalase (CAT), and malondialdehyde (MDA) were detected by using commercial assay kits (Nanjing Jiangcheng Bioengineering Institute, Nanjing, China) according to the operation guide of the kit. The 2,2-diphenyl-1-picrylhydrazyl (DPPH) scavenging activity was noted according to our previous study (16).

### Meat Quality

Six goats per group were slaughtered at the end of the experiment for carcass and the left LD muscle was separated for meat quality examination. The parameters as follows: (1) live weight before slaughter (kg): the body weight was measured after stopping feeding for 24 h and water for 2 h before slaughter; (2) carcass weight (kg): after slaughter, blood, fur, head, hoof, and internal organs (the kidney and kidney fat were retained) were removed, weighing the carcass after standing for 20–30 min; (3) dressing percentage (%): dressing percentage = (carcass weight/live weight before slaughter) × 100; (4) eye muscle area (cm^2^): the cross-sectional area of the LD muscle between the 12^th^ and 13^th^ ribs of the carcass, the outline of the cross section of the eye muscle was drawn with sulfuric acid drawing paper, and then the eye muscle area was calculated by using a digital planimeter KP-90N (No. H18049, made in Japan); (5) pH: the pH value was detected by a pH-star (Matthäus, Eckelsheim, Germany); (6) percentage of water loss (%): a 1 cm thick sample was cut with a 2.5 cm diameter and weighed, then the sample was pressed under 35 kg for 5 min and weighed again, and the percentage of water loss = [(prepressure weight - postpressure weight)/prepressure weight] × 100; (7) drip loss (%): the trimmed meat sample (2.0 × 3.0 × 5.0 cm) was put in a special plastic bag, the bag was inflated and closed, then it was placed in a 4°C refrigerator for 24 h, the juice on the surface of the meat sample was wiped off with filter paper, and the meat was weighed, drip loss = [(initial weight–final weight)/initial weight] × 100%; (8) cooking loss (%): ~100 g of sample (m_1_) was weighed after removing the fascia, epimysium or fat, and the sample was boiled in water for 30 min, cooled for 30 min and weighed (m_2_), cooking loss = (m_1_- m_2_) × 100; (9) shear force (kg): shear force was observed by a digital display tenderness meter (Xielikeji Co., Ltd., Harbin, China) after drilling the muscle column along the direction of the muscle fiber; and (10) meat color (Opto): meat color was measured using an Opto-Star equipment (Company MATTHÄUS, KLAUSA). Each sample was tested in triplicate.

### Fatty Acids

FAs were extracted using a chloroform methanol solution according to Tian et al. (17). Individual FAs were detected using a gas chromatography mass spectrometer (GC–MS; Thermo Fisher Scientific, USA). The GC–MS conditions were as follows: Thermo TG-FAME capillary column (50 m × 0.25 mm × 0.20 μm), 1 μL of injection volume, split ratio of 8:1; injection port temperature of 250°C, ionization temperature of 230°C, transmission line temperature of 250°C, and quadrupole temperature of 150°C. Helium gas was used as the carrier, the flow rate of the carrier gas was 0.63 mL/min, and the energy of ionization was 70 eV.

### Amino Acids

A total 50 mg of meat sample was weighed and transferred to a 2 mL tube. Then, 600 μL of 10% formic acid methanol solution and two steel balls were added and it was run at 60 Hz for 1 min using a high-throughput tissue grinder. Ten microliters of supernatant was added to 490 μL of 10% formic acid methanol solution after centrifugation at 12,000 × g for 5 min at 4°C (Hunan Xiangyi Centrifuge Instrument Co., Ltd., H1850R, Hunan, China) and vortexed at 30 s by a vortex mixer (Haimen Kylin-bell Lab Instruments Co., Ltd., QL866, Jiangsu, China). The 100 μL of diluted sample was mixed with 100 μL of dual isotope internal standard (100 ppb) and vortexed at 30 s. The sample was filtered through a 0.22 μm nylon syringe filter, and the amino acids were detected with a Waters ACQUITY ultra-performance liquid chromatography (UPLC, Waters, Milford, USA) system and tandem mass spectrometry (MS, SCIEX-6500Qtrap; AB Allen-Bradley, Milwaukee, WI) using the internal standard method.

The UPLC conditions were as follows: individual AAs were separated on an ACQUITY UPLC® BEH C18 column (2.1 × 100 mm × 1.7 μm, Waters, Milford, USA) with a column temperature of 40°C; the injection volume was 5 μL; mobile phase: A = 10% methanol (containing 0.1% formic acid) and B = 50% methanol (containing 0.1% formic acid). The gradient elution conditions were as follows: 0~6.5 min, 10~30% B; 6.5~7 min, 30~100% B; 7~8 min, 100% B; 8~8.5 min, 10~100% B; 8.5~12.5 min, 10% B. The flow rate was as follows: 0~8.5 min, 0.3 mL/min; 8.5~12.5 min, 0.3~0.4 mL/min. The MS conditions were as follows: electrospray ionization source, positive ion ionization mode; ion power temperature was 500°C, ion source voltage was 5,500 V; collision gas pressure of 6 psi, curtain gas pressure of 30 psi; nebulization gas pressure and aux gas pressure were both 50 psi; and multiple-reaction monitoring scan mode. Twenty-two amino acids were detected: alanine (Ala), 4-aminobutyric acid (GABA), serine (Ser), glycine (Gly), proline (Pro), valine (Val), threonine (Thr), isoleucine (Ile), leucine (Leu), asparagine (Asn), ornithine hydrochloride (Orn), aspartic acid (Asp), homocysteine (Hcy), glutamine (Gln), lysine (Lys), glutamic acid (Glu), methionine (Met), histidine (His), phenylalanine (Phe), arginine (Arg), tyrosine (Tyr), and tryptophan (Trp).

### Statistical Analysis

The observations were analyzed by using the Statistical Analysis System 9.1.3 software (SAS Institute, Cary, NC, USA) to conduct a one-way analysis of variance model: Y_*ij*_ = μ +τ_*i*_ + ε_*ij*_, where Y_*ij*_ is the observation j (j = 1–6) in the treatment i (i = CON, LS, and HS), μ is the overall mean, τ_*i*_ is the effect of the treatment, ε_*ij*_ is the random error with a mean of 0 and variance σ^2^. The level of significance was set to 0.05.

## Results

### Growth Performance

Adding SY had no significant influence (*P* > 0.05) on the DMI, growth performance (ANG, ADG) or the FCR of the goats (Table 2).

**Table 2:** Effect of purple corn pigment on DMI and growth performance of goats.

**Item^1^**	**Group^2^**			**SEM**	***P*-value**
	**Control**	**LS**	**HS**		
DMI, g/d	816.26	810.22	814.33	6.7455	0.8113
Initial weight, kg	25.45	25.80	26.01	0.6431	0.8231
Final weight, kg	29.80	30.16	30.88	0.6136	0.4875
ANG, kg	4.35	4.36	4.36	0.1692	0.9993
ADG, g	72.56	72.72	72.70	2.8176	0.9990
FCR	11.32	11.32	11.32	0.4567	0.9999

1 *Values represent the mean of six replicates (n = 6)*.

2 *DMI, dry matter intake; ANG, average net gain; ADG, average daily gain; FCR, feed conversion ratio*.

### Antioxidant Activity

The inclusion of HS resulted in significantly higher (*P* < 0.05) levels of muscle TAC, GSH-Px and DPPH scavenging activity than that of the control group (Table 3). In contrast, goats receiving HS exhibited a significant decline (*P* < 0.05) in MDA concentration compared with the control group.

**Table 3:** Effect of selenium-yeast on muscle antioxidant activity and immune of goats^1^.

**Item^2^**	**Group^3^**			**SEM**	***P*-value**
	**Control**	**LS**	**HS**		
TAC, U/mL	4.56^b^	7.36^ab^	9.87^a^	1.0668	0.0348
SOD, U/mL	22.46	22.54	22.89	0.2868	0.5594
GSH-Px, U/mL	170.02^b^	204.14^ab^	238.27^a^	14.9544	0.0489
CAT, U/mL	19.27	19.16	19.10	0.0562	0.2514
MDA, nmol/mL	4.37^a^	1.84^b^	1.28^b^	0.5888	0.0047
DPPH scavenging activity, %	7.36^b^	14.50^a^	14.20^a^	1.1177	0.0312

1a−b *Different letters within a row are significantly different (P < 0.05)*.

2 *Values represent the mean of six replicates (n = 6)*.

3 *TAC, total antioxidant capacity; SOD, superoxide dismutase; GSH-Px, glutathione peroxidase; CAT, catalase; MDA, malondialdehyde; DPPH, 2,2-diphenyl-1-picrylhydrazyl*.

### Meat Quality

As shown in Table 4, the muscle chemical composition was not influenced significantly (*P* > 0.05) by dietary SY supplementation. Similarly, there was no difference (*P* > 0.05) in live weight before slaughter or carcass weight among the dietary treatment groups (Table 5). However, the dressing percentage of goats fed a high SY diet was higher (*P* < 0.05) than that of goats fed a basal or low SY diet. Furthermore, adding a high dosage of SY significantly increased (*P* < 0.05) the eye muscle area of the goats. The lowest shear force was presented by goats fed HS, followed by LS and the control (*P* < 0.05). Dietary treatment did not affect (*P* > 0.05) the pH values (pH_45min_ and pH_24h_), percentage of water loss, drip loss, or cooking loss. Meat color from the goats in the HS group was significantly (*P* < 0.05) darker than that in the other groups.

**Table 4:** Effect of selenium-yeast on muscle chemical composition of goats.

**Item^1^**	**Group**			**SEM**	***P*-value**
	**Control**	**LS**	**HS**		
Moisture, %	73.71	73.90	73.07	0.6206	0.6343
Gross energy, kJ/g	22.39	22.59	22.76	0.2635	0.6371
Crude protein,%	80.97	81.90	80.38	0.9186	0.5131
Ether extract, %	8.87	8.22	8.67	0.9032	0.8755
Ash, %	5.99	6.22	5.84	0.4716	0.8653
Calcium, %	0.33	0.32	0.37	0.0381	0.7202
Phosphorus, %	0.16	0.18	0.18	0.0403	0.9435

1 *Values represent the mean of six replicates (n = 6)*.

**Table 5:** Effect of selenium-yeast on carcass traits of goats^1^.

**Item^2^**	**Group**			**SEM**	***P*-value**
	**Control**	**LS**	**HS**		
Live weight before slaughter, kg	25.60	24.11	26.57	0.7146	0.1245
Carcass weight, kg	11.23	10.48	12.30	0.5345	0.1304
Dressing percentage, %	43.88^b^	43.48^b^	46.23^a^	0.4943	0.0156
Eye muscle area, cm^2^	12.31^b^	11.67^b^	13.99^a^	0.5118	0.0109
pH_45min_	6.03	6.20	5.78	0.1331	0.2338
pH_24h_	5.19	5.40	5.08	0.2572	0.6741
Percentage of water loss, %	3.22	3.50	3.96	0.3894	0.4262
Drip loss, %	1.10	1.44	0.94	0.2418	0.3543
Cooking loss, %	71.96	71.15	66.41	1.9499	0.1285
Shear force, kg	5.42^a^	4.96^b^	4.29^c^	0.1520	0.0004
Meat color, Opto	69.07^b^	70.62^ab^	74.63^a^	1.7376	0.0972

1a−c *Different letters within a row are significantly different (P < 0.05)*.

2 *Values represent the mean of six replicates (n = 6)*.

### Fatty Acids

For the SFA profiles, muscle C11:0 and C21:0 were not detected in either of the two treatments (Table 6). Feeding 4.8 mg/kg SY led to significantly (*P* < 0.05) decreased levels of C8:0, C14:0, C15:0, C16:0, C17:0, C18:0, C20:0 and total SFAs compared with the control group. For the UFA profiles, goats receiving 2.4 mg/kg SY had significantly (*P* < 0.05) increased C16:1, C17:1, C18:1n7, C18:2n6, C18:3n3, C20:4n6, C22:1n9, and PUFA relative to the control group. In addition, supplementation with a high dose of SY (4.8 mg/kg) led to a significant (*P* < 0.05) increase in C15:1 in comparison with that of the control group. Compared to the control group, there were higher (*P* < 0.05) levels of C18:1n9, C22:4, and monounsaturated FAs (MUFAs) in the treatment groups.

**Table 6:** Effect of selenium-yeast on *longissimus dorsi* fatty acid of goats^1^.

**Items^2^, μg/g fresh muscle**	**Group^3^**			**SEM**	***P*-value**
	**Control**	**LS**	**HS**		
**SFA profiles**
C6:0	1.563	1.243	1.225	0.2053	0.5075
C8:0	2.459^a^	1.218^b^	1.138^b^	0.2425	0.0515
C10:0	5.603	6.656	6.423	0.6689	0.5697
C11:0	0.907	ND	ND	-	-
C12:0	3.159	3.253	2.373	0.6081	0.5903
C13:0	3.031	2.668	2.170	0.3273	0.3143
C14:0	239.286^a^	88.203^b^	42.475^c^	14.3397	0.0047
C15:0	20.798^a^	19.151^ab^	16.585^b^	0.6038	0.0356
C16:0	1336.799^a^	1227.075^a^	949.880^b^	52.5783	0.0290
C17:0	54.791^a^	38.120^ab^	33.478^b^	3.7999	0.0564
C18:0	1153.294^a^	1046.130^ab^	824.310^b^	58.8402	0.0615
C20:0	10.090^a^	9.362^ab^	7.549^b^	0.5499	0.0959
C21:0	1.841	ND	ND	-	-
C22:0	1.199	1.111	1.025	0.0782	0.4067
C24:0	0.860	0.556	0.551	0.0991	0.1804
SFA	2832.932^a^	2444.745^a^	1889.183^b^	96.8306	0.0143
**UFA profiles**					
C14:1	12.233	12.769	12.408	0.8436	0.9033
C15:1	3.164^b^	3.765^ab^	4.251^a^	0.1156	0.0159
C16:1	33.416^c^	74.780^a^	60.249^b^	1.2307	0.0004
C17:1	11.880^b^	26.373^a^	18.840^ab^	1.6770	0.0203
C18:1n7	45.507^b^	79.469^a^	52.032^b^	2.3755	0.004
C18:1n9	643.434^b^	1053.533^a^	1199.596^a^	36.9793	0.0037
C18:1n12	629.701	672.080	691.810	73.6957	0.8396
C18:2n6	160.463^b^	239.745^a^	164.527^b^	10.2805	0.0200
C18:3n3	8.375^b^	16.063^a^	10.766^ab^	1.5727	0.0850
C20:1	9.409	10.515	6.790	0.9903	0.1535
C20:2	3.811	3.889	2.490	0.5603	0.2842
C20:3n6	15.489	15.539	12.191	1.5585	0.3508
C20:4n6	193.545^b^	242.500^a^	201.060^ab^	9.4848	0.0656
C20:5n3	6.399	8.519	6.880	0.8689	0.3308
C22:1n9	67.442^b^	84.764^a^	66.917^b^	2.4024	0.0215
C22:4	22.019^b^	28.588^a^	28.423^a^	0.7843	0.0153
C22:5n3	28.127	31.578	28.668	1.1157	0.2084
C22:5n6	5.571	4.996	4.982	0.2241	0.2531
C22:6n3	4.662	4.171	4.819	0.7170	0.8129
C24:1	13.752	12.300	11.358	11.358	0.2210
MUFA	1469.939^b^	2030.351^a^	2124.252^a^	47.4271	0.0043
PUFA	448.460^b^	595.589^a^	464.807^b^	9.9248	0.0033

1a−c * Different letters within a row are significantly different (P < 0.05)*.

2 *Values represent the mean of six replicates (n = 6)*.

3 *ND, not detected; SFA, sum of all the saturated fatty acid; MUFA, sum of all the monounsaturated fatty acid; PUFA, sum of all the polyunsaturated fatty acid*.

### Amino Acids

As shown in Table 7, there were no significant differences (*P* > 0.05) in muscle Ala, Pro, Thr, Asn, Lys, His, Arg, Trp, total AA (TAA), or essential AA (EAA) values among the three groups. Moreover, Gly and Hcy were unable to be detected in all groups. Compared to the control, feeding with 2.4 mg/kg SY induced significant (*P* < 0.05) increases in GABA, Glu and umami amino acid (UAA) concentrations. Furthermore, the HS group had significantly higher (*P* < 0.05) muscle Ser, Val, Ile, Leu, Orn, Met, and Tyr levels than the control group.

**Table 7:** Effect of selenium-yeast on *longissimus dorsi* amino acid of goats^1^.

**Item^2^, μg/g fresh muscle**	**Group^3^**			**SEM**	***P*-value**
	**Control**	**LS**	**HS**		
Ala	499.99	476.86	535.56	13.3912	0.1141
GABA	2.97^b^	6.33^a^	1.51^b^	0.5517	0.0183
Ser	128.68^b^	144.50^ab^	153.42^a^	4.0041	0.0484
Gly	ND	ND	ND	-	-
Pro	33.86	37.72	37.68	5.1362	0.8386
Val	63.35^b^	54.94^b^	84.91^a^	4.2188	0.0319
Thr	38.44	39.49	46.95	3.6363	0.3319
Ile	24.59^b^	23.84^b^	42.56^a^	3.6072	0.0569
Leu	59.89^b^	52.26^b^	78.13^a^	3.4790	0.0285
Asn	607.13	590.97	646.23	33.8619	0.5615
Orn	13.09^b^	23.72^ab^	31.95^a^	2.7746	0.0387
Asp	27.55^ab^	38.00^a^	14.64^b^	3.5104	0.0410
Hcy	ND	ND	ND	-	-
Gln	2105.77^ab^	2464.45^a^	2029.24^b^	83.5823	0.0655
Lys	137.13	121.46	121.08	13.0921	0.6546
Glu	127.67^b^	240.74^a^	94.34^b^	19.4901	0.0262
Met	22.69^b^	21.50^b^	32.07^a^	1.8712	0.0499
His	656.24	476.20	690.34	107.5521	0.4272
Phe	30.06^ab^	25.91^b^	40.75^a^	2.5850	0.0558
Arg	91.38	87.72	78.34	5.0183	0.3072
Tyr	43.68^b^	36.64^b^	72.02^a^	3.5099	0.0112
Trp	8.09	7.74	12.05	1.1869	0.1398
TAA	4722.26	4970.99	4843.77	173.0152	0.6414
EAA	384.25	347.14	458.49	29.3495	0.1535
UAA	728.95^b^	818.15^a^	757.32^ab^	15.5887	0.0477

1a−b * Different letters within a row are significantly different (P < 0.05)*.

2 *Values represent the mean of six replicates (n = 6)*.

3 *ND, not detected; Ala, alanine; GABA, 4-aminobutyric acid; Ser, serine; Gly, glycine; Pro, proline; Val, valine; Thr, threonine; Ile, isoleucine; Leu, leucine; Asn, asparagine; Orn, ornithine hydrochloride; Asp, aspartic acid; Hcy, homocysteine; Gln, glutamine; Lys, lysine; Glu, glutamic acid; Met, methionine; His, histidine; Phe, phenylalanine; Arg, arginine; Tyr, tyrosine; Trp, tryptophan; TAA, total amino acid; EAA, essential amino acid (Val, Thr, Ile, Leu, Lys, Met, Phe, Trp); UAA, umami amino acid (Gly, Ala, Asp, Glu, Phe, Tyr)*.

## Discussion

Small ruminants are more prone to OS status, resulting in reduced growth performance and impaired immunity, leading to depressed husbandry efficiency (18). Hence, finding a suitable method to improve the antioxidation ability of small ruminants and eliminate free radicals (FRs) is a hot subject. Se is not produced by the body and must be obtained through food and water (19). Dietary Se supplementation is the most common method to improve the Se status of ruminants. Se is an essential trace element in biological life activities, playing a biological function in the form of selenoproteins, such as GSH-Px, thioredoxin reductases, deiodinases, and selenoproteins; alternatively, Se exerts its biological function when incorporated into proteins as selenocysteine residues, replacing the sulfur moiety in cysteine (20). Thus, Se, as a component of inactivators of toxic heavy metals, plays an important structural and enzymatic function, able to neutralize ROS and enhance antioxidant activity, protecting against lipid oxidation in the ruminant body (21). Indeed, Se can improve the antioxidant defense system in the body by modulating ROS-generating enzymes and additional synthesis of antioxidant enzymes and promoting inflammation in a cooperative and interactive way, being critically important for the ruminant's adaptation to nutritional stress (22).

Chauhan et al. (23) reported that Se supplementation could improve antioxidant enzymes in the body, protecting against OS and improving ruminant health. Vignola et al. (7) showed that supplementation with Se can enhance muscle Se levels, reducing the lipid oxidation of lambs. Thus, adding SY to the diet could strengthen antioxidant activity in muscle in this study, perhaps because Se is a part of the active center of GSH-Px, which can catalyze the reduction of peroxides (24). This may also be attributed to dioxide Se being a source of oxidant, which can oxidize some groups, such as hydroxides and methyl groups, to aldehyde groups and is reduced to Se itself during the chemical reaction (25). Consistent with our conclusions, Alimohamady et al. (26) showed that dietary Se supplementation did not impact the DMI, ADG, and FCR, whereas it can improve GSH-Px activity in lambs. Similar values of antioxidant activity in the body of fattening lambs receiving Se were obtained by Antunović et al. (4).

Meat quality traits include color, texture, hardness, tenderness, marble, etc. Among them, the amount of fat deposition and depositional mode are very important. The dressing percentage is an important index to evaluate carcass quality. Generally, the larger the eye muscle area is, the higher the lean meat rate (27). Netto et al. (28) showed that Se supplementation altered lipid metabolism in confined Brangus cattle by reducing the cholesterol concentration in the meat. Similarly, Se intake led to altered mRNA expression of genes associated with cholesterol and lipid metabolism parameters in the liver and LD muscle of growing Polish Merino lambs (29). In the current study, the addition of high SY to the diet improved the dressing percentage and eye muscle area, which may be related to Se regulating fat deposition by regulating lipid metabolism parameters in ruminants. Tenderness is critically important from a sensory viewpoint. Shear force is a measurement that indicates tenderness, mechanically assessed through the force necessary to sever the muscle fibers (30). Meat tenderness is among the important determinants of sensory quality parameters associated with consumer preferences. In the present study, goat meat from the SY diet had a lower shear force value than that from the control diet, indicating a tender texture. Meat is primarily comprised of myoglobin (Mb). The O_2_ molecule can be bound to Fe^2+^ and is stabilized through hydrogen binding by the nearby distal His (31). Hence, an important reason for the meat color changing is because the Fe^2+^ in Mb is unstable, and it is easy to oxidize to Fe^3+^, resulting in discoloration of the product (32). Therefore, supplementation with SY could improve the meat color of goats, suggesting that Se may prevent the oxidation of Fe^2+^ to Fe^3+^, thus improving the meat color.

It is well-known that O_2_ is vital for all animals. However, some O_2_ is converted to superoxide (${\text{O}}_{2}^{-}$) and then converted into hydrogen peroxide (H_2_O_2_) and H_2_O by SOD, GSH-Px, and CAT enzymes (33, 34). FR scavengers are substances that can scavenge FR or block FR involved in oxidative reactions. Additionally, most FR scavengers are antioxidants, which can control the formation of FR by reducing the concentration of active FR intermediates and reducing the efficiency of the expansion stage in the FR chain reaction. UFAs in lipids are active in chemical activities because they contain multiple double bonds, very susceptible to destruction by FR and peroxidation (35). Lipid peroxidation in UFAs is a reaction where FR removes an electron from the lipids because molecular O_2_ is incorporated into unsaturated lipids to form a lipid hydroperoxide (36). Hence, lipid peroxidation is a natural phenomenon involved in peroxidative loss of unsaturated lipids, thus causing lipid degradation and membrane disorders (37). Ruminant meat is characterized by a high SFA content and a low PUFA content due to biohydrogenation of UFAs in the rumen by microflora (38). Purba et al. (39) did show that SFAs are showed in higher levels compared to the PUFAs in ruminant meat. In biological systems, lipid peroxidation is controlled by antioxidant defense systems that include antioxidant nutrients. Although Se is not an antioxidant, it is necessary for the production of several enzymes that affect the antioxidant network. Additionally, Se exists in the cytoplasm and mitochondrial matrix, which can reduce toxic peroxides to non-toxic hydroxyl compounds and decompose H_2_O_2_ into alcohols and water (40). Noticeably, Se also has a synergistic effect with vitamin E, which means that the two nutrients are more powerful than either one alone (19). Kišidayová et al. (41) demonstrated that SY could impact the intake of dietary PUFAs by rumen microbes, especially rumen ciliates, as well as the biohydrogenation of PUFAs to more SFAs. Niedwiedzka et al. (42) suggested that feeding SY can improve the muscle MUFA and PUFA ratios and increase the concentration of EAAs. Thus, Se has a strong antioxidant function, which can improve the content of beneficial FAs in animal byproducts by regulating the hydrogenation of PUFAs in the rumen.

Low intake and poor utilization of feedstuffs could be negative effect on the rumen ecosystem and rumen fermentation (43, 44). Adding of SY did not differ on the DMI and growth performance, while it could improve antioxidant activity in goat meat. Moreover, Ferreira et al. (45) who showed that Se supplementation did not improve rumen fermentation and nutrient utilization, but it tended to increase the concentration of PUFA in omasum flow of FAs. Thus, we observed that SY could improve the meat UFA concentrations of goats in the present study. The mechanism may be related to increasing the antioxidant activity and inhibiting the production of FR, resulting in a reduction in the lipid peroxidation reaction. In addition, feed composition affects ruminal fluid fermentation (46, 47), the affinity of rumen microorganisms (48). Dietary supplementation of SY may improve UFA concentrations by altering rumen fermentation model and ruminal microbiota in goats. Further studies are needed to observe the effect of SY on ruminal microorganisms. This is consistent with the results of Aghwan et al. (49), who demonstrated that supplementation with Se can lead to an increase in UFAs in the supraspinatus, *longissimus lumborum*, and *semitendinosus* muscles in Kacang goats. Yong et al. (50) also had a similar observation, that Korean native Hanwoo cattle fed a diet supplemented with organic Se had improved growth performance, carcass traits, and meat quality with an enriched PUFA profile. However, Pereira et al. (51) found that Nellore steers receiving Se did not have any changes in the FA profile of their *LD* muscle. The reason for the different observations may be as follows: (1) adding different SYs as the source of Se to the daily diet; (2) different species of animals have different metabolic pathways of Se; and (3) different Se compounds and their structural formulas could also be contributing factors (52, 53).

When the body is in a state of OS, it may participate in the AA metabolic process because ROS can also oxidize blood and structural proteins and inhibit the proteolytic system (54). Dai et al. (55) showed that cysteine limitation reduces the biosynthesis of glutathione (GSH) and causes the depletion of GSH, which directly inhibits the activity and stability of GPX4. In addition, cystathionine, cysteine and glycine are important precursors in the synthesis of GSH-Px, which has a rate-limiting role in the synthesis process (56). Indeed, AA has been shown to regulate the upstream glutathione synthesis pathway by reducing OS and the subsequent inflammatory response in ruminants (57). Specifically, most of the Se in the body exists in the active center of specific selenoproteins/selenases in the form of selenocysteine, is non-specifically incorporated into ordinary proteins in the form of selenomethionine, and it exists in the form of methylated Se compounds (58). In this study, Se cleared the FR in goats and upregulated the OS status of the body, thereby improving muscle AAs. Additionally, protein oxidation can cause AA loss or they can be fragmented, leading to alterations of structural proteins or alterations of enzyme functions (54). This is perhaps because Se in the liquid phase binds to protein, followed by microbial cell protein hydrolysis, and then it is absorbed as a Se-containing free AA (59). Of interest, the AA content increased with supplementation with a high dose of SY in this study. One possible reason is that ruminal bacteria are capable of utilizing Se from selenite and selenate for the synthesis of selenoamino acids (60). For young ruminants, Se is closely linked with vitamin E and sulfur-containing AAs (61). Gressley (62) who found that Se is covalently bound to AA to form AA complex trace minerals, with increased bioavailability in ruminants, suggested that allows more AA to be deposited in muscle. This might also be the reason for the lower apparent Se absorption by ruminants than by monogastric animals.

The types of AA can be divided into tasteless, bitter, sweet, and umami amino acid according to taste characteristics. The UAAs are represented by Gly, Ala, Asp, Glu, Phe, and Tyr. When the expression of umami substance regulatory genes is high, the content of umami substances in the meat is also increased (63). In addition, adenylosuccinate lyase (ADSL) plays a key role in the formation of umami substances. Interestingly, muscle tenderness can be affected by cellular antioxidants, and ADSL has been previously identified as a putative biomarker of tenderness, being positively correlated with beef tenderness (64). In the current study, SY improved UAA in the LD muscle of goats. Regarding the possible mechanism, it could be assumed that Se can prevent excessive FR, enhance antioxidant enzymes and modulate ADSL-related umami gene expression in the body, thus leading to an increase in UAA content in the muscle of goats, but this needs further verification. Additionally, the meat is rich in Se concentration, the integrity of cell membrane is protected after slaughter, reducing the loss of umami substances, and thus improving UAA content in LD muscle in goats. In short, the experimental results demonstrated that SY supplementation may be useful for growing goats because of its high antioxidant potential.

## Conclusion

The findings of the current study suggest that dietary Se supplementation could not only improve the muscle antioxidant activity and meat quality but also improve the amino acid and fatty acid profiles of meat from Qianbei-pockmarked goats. Additional studies are required to clarify the effects of SY on the conjugated PUFA concentrations in the rumen microorganisms of goats from transcriptomics and metagenomics.

## Data Availability Statement

The raw data supporting the conclusions of this article will be made available by the authors, without undue reservation.

## Ethics Statement

The animal study was reviewed and approved by the Experimental Animal Ethics, Guizhou University (Guiyang, China).

## Author Contributions

X-ZT: data curation, writing-original draft preparation, investigation, and data curation. J-XL, Q-YL, XW, and M-MX: investigation and data curation. DZ: supervision. QL: methodology and writing-reviewing and editing. XC: project administration and supervision. All authors have read and approved the final manuscript.

## Funding

The research was funded by the Science and Technology Project of Guizhou Province (Qiankehe foundation-ZK[2021] General 164), the National Natural Science Foundation of China (31760652), the Cultivating Project of Guizhou University (2019-33), the Start-up Fund of Guizhou University (2016-76; 2019-26), and the Core Program of Science and Technology Department of Guizhou Province (QKH[2019]2279), respectively.

## Conflict of Interest

The authors declare that the research was conducted in the absence of any commercial or financial relationships that could be construed as a potential conflict of interest.

## Publisher's Note

All claims expressed in this article are solely those of the authors and do not necessarily represent those of their affiliated organizations, or those of the publisher, the editors and the reviewers. Any product that may be evaluated in this article, or claim that may be made by its manufacturer, is not guaranteed or endorsed by the publisher.
